# Art Therapy and Art Making for Addressing Cancer-Related Pain and Distress in Adult Populations: A Scoping Review

**DOI:** 10.3390/ijerph22121877

**Published:** 2025-12-17

**Authors:** Nina H. Russin, Alexis M. Koskan, Matthew P. Martin

**Affiliations:** College of Health Solutions, Arizona State University, Health North, 550 N. 3rd St., Phoenix, AZ 85004, USA

**Keywords:** art therapy, art making, cancer pain, emotional distress

## Abstract

**Highlights:**

**Public health relevance—How does this work relate to a public health issue?**

- Cancer is a leading cause of morbidity and mortality globally, with over 20 million new diagnoses annually, and is expected to increase to over 35 million new cases annually, according to the World Health Organization (2024).
- Moderate-to-severe pain and associated emotional distress affect up to 40% of cancer survivors, significantly affecting quality of life.

**Public health significance—Why is this work of significance to public health?**

- While pharmacotherapy remains a standard treatment for cancer pain management, art therapy and art making as adjunct therapies may help cancer survivors self-manage subjectively rated chronic pain, stress, anxiety, and depression.
- Art making and art therapy are culturally competent approaches to chronic pain self-management among underserved populations, including Indigenous communities.

**Public health implications—What are the key implications or messages for practitioners, policy makers, and/or researchers in public health?**

- Art making and art therapy may be part of a holistic, multimodal approach to cancer treatment, which puts the patient at the center of the care team.
- Neuroimaging research has linked aesthetic experiences with activation of the brain’s “reward network,” providing a physiological basis for engaging with art as a strategy for self-management of chronic pain.

**Abstract:**

Background: Worldwide, cancer is a leading cause of morbidity and mortality, with symptoms of pain and emotional distress, associated with the disease and its treatment. Art therapy and art making are promising adjuncts to pharmacotherapy for these symptoms. However, current studies do not support replacing pharmacotherapy with these methods. Research Question: Is there evidence supporting the use of art therapy and/or art making interventions for managing cancer-related pain (primary outcome) and emotional distress (secondary outcome) among adult cancer survivors, during and following active treatment? Methods: We searched six databases, including PubMed, CINAHL, Scopus, ProQuest (library’s version), and Google Scholar, using the search terms “cancer pain” AND “art therapy” OR “art making.” Inclusion criteria included English language, peer-reviewed studies, on adult cancer survivors. The search yielded 1305 results, with 23 meeting the inclusion criteria. Because emotional distress was frequently discussed in the context of cancer-related pain in the included studies, it was added as a secondary outcome. Results: The efficacy of art therapy/art making to manage cancer-related pain and emotional distress was difficult to determine due to the heterogeneity of study designs and interventions. Of the studies reviewed in which pain was a primary outcome, eight found significant pain reductions, three found small or no effects, and three reviews described art making as a non-verbal method of communicating about pain, but did not address changes in pain levels. The terms “art therapy” and “art making” were sometimes used interchangeably. The choice of therapeutic approach was sometimes financially driven, and was also impacted by the availability of certified art therapists. Discussion: Methodological shortcomings of the existing research include small sample sizes, lack of standardized intervention protocols, and inconsistent outcome measures, underscoring the need for more rigorous and generalizable studies. Future research should consider neuroimaging evidence linking aesthetic experiences with activation of the brain’s “reward network” by utilizing fMRI to study brain activity during art therapy and art making interventions.

## 1. Introduction

Cancer is a leading cause of morbidity and mortality, with global estimates of 19.3 million new diagnoses in 2020 and 20 million new cases in 2022, estimated to increase to 35 million new cases annually by 2050 [1,2]. Pain is one of the most feared symptoms among cancer survivors, due to both its impact on survivors’ quality of life and because many believe that cancer-related pain signals disease recurrence [3]. Nearly 40% of all cancer survivors endorse moderate-to-severe pain, with 55% of patients receiving cancer treatment and 66.4% of patients with advanced cancer reporting pain [4]. In addition, many cancer survivors report associated emotional distress [5,6,7,8], which contributes to the significant effect that disease progression and its treatment have on quality of life.

Cancer pain is challenging to control due to the different sources of pain (tumor invasion, treatment side effects, etc.) and types of pain, including both chronic and acute episodes of breakthrough pain [9]. While pharmacotherapy utilizing opioids and/or nonsteroidal anti-inflammatory drugs (NSAIDs) remains a standard of care, there are numerous reports of their limited efficacy and concerns regarding tolerance and medication side effects [10]. These challenges have fueled the growing interest in complementary alternative modalities (meditation, massage, acupuncture) and lifestyle strategies as adjuncts to allopathic medicine.

Two promising approaches for the management of cancer pain and emotional distress are art making and art therapy. According to the American Art Therapy Association (AAAT), art therapy is “an integrative mental health and human services profession that enriches the lives of individuals, families and communities through active art making, creative processes, applied psychological theory and human experience within a psychotherapeutic relationship,” [11]. Today the term art therapy connotes a variety of approaches ranging from psychoanalytic methods rooted in Freudian theory to so-called “open studio”: communal art making in non-clinical settings [12]. In the U.S., art therapy is delivered by registered art therapists, a vocation that requires a master’s degree in art therapy, supervised practicum and internship, post-educational clinical experience as a provisional registered art therapist, and certification by the Art Therapy Credentials Board [13]. By comparison, art making behavioral interventions are delivered by non-certified clinical staff members or community art educators.

Because of its relatively low cost and ease of integration into pain and emotional distress management strategies for cancer survivors, interest in utilizing various forms of art therapy and art making is increasing globally. Given the variety of interventions that have been conducted globally, a scoping review is warranted to map the evidence and synthesize outcomes across diverse populations and settings. To date, studies tend to be limited to small sample sizes, with mixed results as to efficacy. Complicating the picture, various studies have utilized different measures for patient self-report of pain and emotional distress [5], with the Edmonton Symptom Assessment Scale (ESAS), Spielberger State–Trait Anxiety Index (STAI-S) and WHO Quality of Life (WHOQOL-BREF) being among the more popular [8,14]. In addition, studies are heterogeneous in terms of intervention settings (inpatient, outpatient, and community) and cancer diagnosis, with some studies not listing a specific diagnosis or staging.

Support for continued research in this area may come from recent neuroimaging research, linking empirical studies of the brain to behavior [15]. Functional magnetic resonance imaging (fMRI) and positron emission tomography (PET) have confirmed that beautiful objects may stimulate the mesolimbic dopamine system and associated brain reward circuitry. The roots of this lie in human evolution: the brain is motivated to prioritize aesthetically pleasing stimuli that signal safety, resource acquisition, and social cohesion, reinforcing behaviors critical to survival and overall health. In the case of aesthetically beautiful objects, this reward system receives input from the vision system (particularly V1 and V4), which, in turn, activates the anterior cingulate cortex, orbital frontal cortex, striatum, amygdala, and insula. Areas of the brain within this “reward network”, including the nucleus accumbens and ventral tegmental area, also control the release of dopamine, endogenous opioids, and endogenous cannabinoids, all of which are known to ameliorate painful sensations and contribute to feelings of pleasure [16]. These findings support the idea that engaging with art, whether by viewing artworks or creating them, is intrinsically rewarding and may help to manage pain and emotional distress. Studies that utilize neuroimaging to collect evidence of brain activity during art therapy/art making may be an important area for future research.

## 2. Research Question

Is there evidence supporting the use of art therapy and/or art making interventions for managing cancer-related pain (primary outcome) and emotional distress (secondary outcome) among adult cancer survivors, during and following active treatment?

## 3. Objectives

To systematically explore the use of art making and art therapy in managing cancer pain and its comorbidities, we conducted a scoping review. The review aims to map and synthesize the existing literature on the use of art therapy and art making in the management of cancer-related pain and emotional distress, defined as stress, anxiety, and depression. Because art therapy and art making interventions may utilize similar techniques, we will attempt to clarify any differences as revealed by these studies. The review concludes with recommendations for art making and art therapy interventions drawn from the literature, and the potential for emerging neuroimaging research on the brain’s “reward network” to contribute empirical data as to the efficacy of these approaches in cancer pain and emotional distress.

## 4. Methods

This scoping review follows the PRISMA 2020 guidelines for scoping reviews (see Supplementary Material File S1, reported on page 13). The review utilizes the PCC framework (population, concept, and context), endorsed by JBI, as a guide to construct a clear and meaningful title for the review. Our review takes a population health approach to evaluating research on the use of art therapy and art making for managing cancer pain and emotional distress. Because reporting of these symptoms is largely subjective, it is important to consider the context (patient’s stage of treatment, clinical settings, and cultural factors), which may impact these ratings. For example, cancer pain among Indigenous cancer survivors is conceptualized differently than for Indo-European cancer survivors, due to the collectivistic culture of the first group versus the individualistic orientation of the second [17,18]. In addition, concepts of art therapy and art making vary widely by location and country due to the availability of art therapy training and certification, and considerable variation within art therapy concepts. Children are not included in this review due to differences in how they express cancer pain compared to adults, particularly non-verbal behavior, as compared to adults verbally describing pain symptoms [19].

### 4.1. Search Strategy

In February 2025, searches were conducted in PubMed, Scopus, CINAHL, ProQuest (library’s version), and Google Scholar using the search terms (“cancer pain” AND “art therapy” OR “art making”). See Appendix A for a specific search string example. No lower date limit was set, and a peer review limit was selected when possible. Included studies employ various art making strategies, ranging from manualized strategies such as Mindfulness-Based Art Therapy (MBAT) and the Body Outline to unsupervised art making activities among cancer survivors in the community. The search strategy was intentionally focused on the precise intersection of “cancer pain” and “art therapy”/“art making” to maximize the relevance of the extracted evidence. This specific approach was essential to maintain the review’s scope, excluding the literature on related modalities (e.g., music or drama, which fall under the broader category of “creative arts”) or studies where pain was not the primary focus (e.g., studies using broad terms like “distress”). While maximizing relevance, we acknowledge that this deliberate specificity may limit the scope of captured evidence, with mapping representing high-specificity islands, as opposed to the entire landscape of available evidence. A science librarian at the university reviewed the search strategy and made suggestions for clarification.

### 4.2. Eligibility Criteria

Inclusion criteria included English-language, peer-reviewed articles describing visual art therapy or art making for managing cancer-related pain in adult (ages 18+) cancer survivors. Authors also made use of two textbooks in the field for information on art therapy history and methods, and recent advances in empirical aesthetics. Exclusion criteria included articles published in languages other than English; studies of pediatric populations; studies utilizing music, drama, or literature interventions that did not include visual art therapy, art making, or art therapy for non-cancer pain; and articles about art therapy or art making that did not address cancer-related pain as an endpoint.

### 4.3. Study Selection Procedure

**Figure 1:**
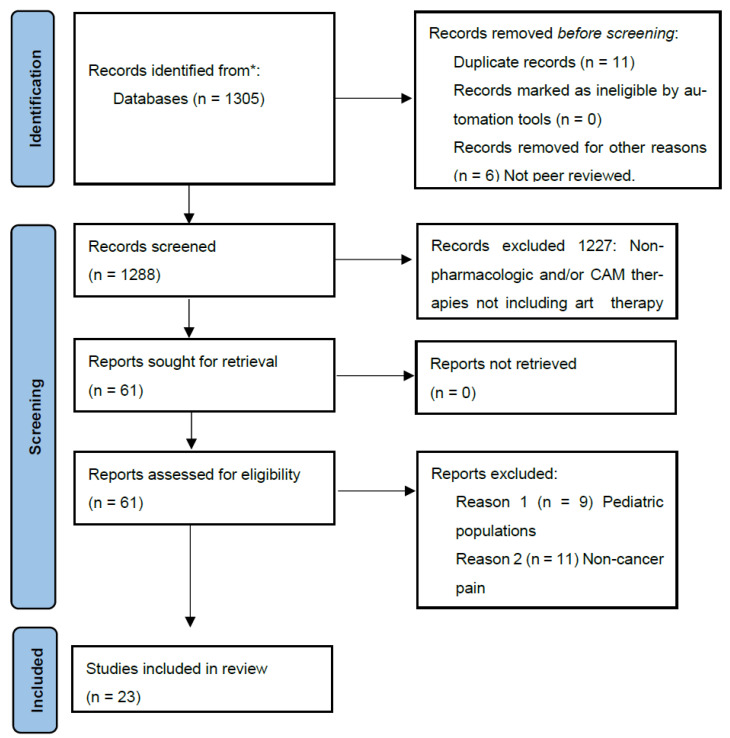
PRISMA flow diagram of study selection. * Complementary alternative medical therapies that were not considered here include the following: acupuncture, active release therapy, art therapy not including a visual art component (music, drama, literature, film, etc.), massage, traditional healing techniques, and meditation therapies that do not include an art making or art therapy element such as ACT, present-moment awareness, attention shifting, etc.

We obtained 1305 results, including 11 duplicates and 6 results that were either not peer-reviewed or not available through the university library (see Figure 1). We reviewed results by title and abstract to determine which addressed subjects directly relevant to the study objectives. For example, we eliminated articles that did not include visual art interventions, but included studies that utilized visual art as part of multimodal (music and art) therapeutic approaches. Finally, authors screened full texts of extracted articles for study design, quality of evidence, and relevance.

### 4.4. Data Extraction and Charting

Data extracted included study design, sample size, intervention type, outcomes related to pain and emotional distress, and setting. Data were organized within an Excel spreadsheet and are summarized in Table 1 and Table 2.

## 5. Results

Twenty-two records fit the inclusion criteria for this study. Selected records included four randomized controlled trials, three systematic reviews, a mapping review, a scoping review, and an integrative review, with the remaining observational, mixed methods, and pilot studies (see Table 1). Table 2 describes the types of art therapy or art making interventions utilized, the setting and duration of therapy, types of therapy providers, and outcomes, as described by the researchers. Specific cancer diagnoses are included when available in the studies reviewed.

### 5.1. Lack of Consensus on Primary and Secondary Outcomes

Authors of systematic, scoping, and mapping reviews cited the heterogeneity of art therapy and art making interventions, small sample sizes, and lack of controls in many studies as making it difficult to compare outcomes across studies and evaluate the efficacy of art therapy and/or art making. None of the reviews mentioned a statistically significant difference in either subjective pain ratings or reductions in emotional distress, although all of the studies described the potential benefits of integrating art therapy into clinical practices. A systematic review evaluating the effects of art and music therapy on breast cancer patients [5] concluded that while these modalities have the potential to ameliorate pain and related emotional distress, the lack of study controls as well as standards guiding the implementation of art therapy made it difficult to quantify its efficacy. A second systematic review [35] mentioned small sample sizes, with 4 of the 13 studies reviewed utilizing samples of 20 individuals or fewer. The efficacy of art therapy to reduce cancer-related pain varied among studies reviewed, with some finding statistically significant reductions in pain, while others found no effect at all. A third systematic review [14] noted that while there were some indications suggesting art therapy could improve quality of life (and related psychosomatic symptoms), randomized controlled clinical trials with larger samples and control groups would be needed to draw generalizable conclusions. Finally, a scoping review [25] found that drawing therapy significantly alleviated pain, fatigue, and chemotherapy-induced nausea, but suggested that standardized intervals between art therapy interventions, as well as improved collaboration between nurses and art therapists, could promote more comprehensive implementation of art therapy in inpatient settings.

Inconsistent use of quantitative measures may have contributed to variability in the reported outcomes. A total of 28 measures (see Appendix B) were cited in 17 of the studies included herein that included quantitative data. Six of the studies [17,18,23,26,29,32] were strictly qualitative. In the case of two studies with Indigenous populations [17,18], avoiding such measures reflects cultural competence, with the research subjects having a strong preference for storytelling and drawing to express both physical and existential pain. The Body Outline study [29] utilized drawing on a life-size body image to both express physical pain and measure its intensity qualitatively. The final three [23,26,32] evaluated pain utilizing thematic analyses and patient interviews. Insights from these studies included the benefit of art making to shift attention away from pain by focusing on life experience outside of cancer [22,32], that art making reduces stress, which is known to exacerbate physical pain [26], and that art making can give symbolic expression to the fear and grief associated with a cancer diagnosis, reflected in existential and physical pain. A mixed methods study conducted with 12 patients with advanced cancer [33] mentioned attention shifting during art making as an effective strategy for managing pain, with patients mentioning that art making helped them to remove themselves mentally from the hospital environment and feel actively engaged with the world again.

The observational studies involving Native American cancer survivors and their caretakers [17,18] utilized digital storytelling interventions to identify needs among cancer caregivers in First Nations communities [17] and drawings as a method for Indigenous cancer survivors to express their subjective feelings of pain [18]. In both cases, the use of visual media was seen as a more effective method of communication than language, since English may be a second language for these individuals. These studies are significant for their methodology: utilizing art to bridge communications gaps between medical professionals and Indigenous individuals from collectivistic cultures, with beliefs about spirituality and healing that are vastly different from traditional Western medicine. As stated by Hodge and colleagues, such communication gaps can lead to undertreatment of pain, reflecting implicit biases among providers, particularly in acute care [18].

Across three multiple study reviews [20,23,25], only one [20] specifically focused on the efficacy of art therapy using a psychotherapeutic approach on patients with advanced cancer. While the authors found significant improvements in pain, depression, and anxiety using validated instruments, they reiterated concerns about the quality of evidence due to the absence of standardized protocols for art therapy interventions, lack of controls, and small samples. Two reviews [23,25] investigated art making within different theoretical frameworks: the first employed Kaplan’s Attention Restoration Theory (ART), which posits that activities linked to fascination (e.g., art making) are restorative, and the second utilized free drawing exercises to address cancer-related pain, stress, anxiety, depression and quality of life. The authors suggested that art making can improve subjective pain ratings by shifting the pain away from the illness, improve self-efficacy by enabling feelings of choice and control, decrease depression, and enhance socialization and quality of life.

### 5.2. Randomized Controlled Trials in Breast Cancer Patients

Four randomized controlled trials [7,21,24,30] studied the use of art therapy in breast cancer patients for improving physical and psychological symptoms associated with the disease and its treatment. Two of the trials [24,30] evaluated the use of Mindfulness-Based Art Therapy (MBAT): using a combination of meditative practices and drawing. Both studies found MBAT successful in addressing stress, depression, anxiety, and health-related quality of life. Although these studies mention improved body awareness and self-regulation, they do not specifically address cancer-related pain. The third study [7] utilized a combination of visual art making (marbling and ney music) and reported decreases in subjectively rated pain, nausea, and anxiety. The fourth study [21] utilized two drawing interventions—visualization of feelings in response to hearing read words and filling in a life-size body outline [29]—finding that participants in the art therapy arm scored lower in measures of depression, anxiety, and somatic symptoms over time, as compared to controls.

### 5.3. Crossover Studies

A crossover study [27] that tested the efficacy of tile painting (Tiles of Hope program) among blood and marrow transplant recipients in an urban outpatient cancer center found significantly reduced symptoms of fatigue, difficulty concentrating, shortness of breath, and pain compared to the control condition. The Tiles of Hope program at the University of Kansas Cancer Center’s outpatient blood marrow transplant unit is staffed on a volunteer basis by occupational therapy faculty and students. Puig et al.’s crossover study [31] employed drawing and painting art therapy interventions in outpatient settings with early-stage breast cancer patients, finding statistically significant improvements in mood (anxiety, depression, fatigue, and confusion, but not for spirituality.

### 5.4. Single Arm Studies

Nine of the studies reviewed were single-arm designs, including four pilot studies conducted in inpatient and outpatient settings [22,26,33,34]. Pilot studies utilized protocols delivered by professional art therapists, with a variety of measurement methods (ESAS, VAS, numerical rating scales, STAI) to evaluate changes in subjective pain, depression, anxiety, stress, and fatigue following the interventions. All studies reported improvements in pain and stress. However, these studies should be considered in the context of small sample size, no control groups, and predominance of female subjects.

Three mixed methods studies utilized art therapy in patients undergoing active treatment [8,28] or in palliative care [6] to study ratings of pain, fatigue, depression, and anxiety using the ESAS and other validated measures, along with semi-structured interviews. All studies primarily enrolled female subjects. Findings were positive for reductions in depression and anxiety, with two of the studies [6,8] also reporting reductions in subjective pain. The predominance of female subjects in these studies is a confounder when considering the efficacy of art therapy and art making for managing cancer-related pain and emotional distress, given research that male and female cancer patients experience pain differently [36,37], with female patients generally having greater pain sensitivity and greater levels of chronic pain. In addition, pain sensitivity among female patients of childbearing age may be influenced by their menstrual cycles.

## 6. Discussion

This study is unique for its inclusion of a range of art therapy and art making interventions related to cancer pain and emotional distress in both clinical and community settings, particularly studies focused on Indigenous cancer survivors and their caretakers. These communities have been largely overlooked in similar studies. Given that Indigenous cultures endorse storytelling and art making as methods of communicating about pain and distress, this is an important area for future research.

Another unique aspect of this review is the suggestion of a physiological basis for the benefits of art therapy/art making based on neuroscientific imaging (fMRI) studies that demonstrate engaging with art stimulates structures within the brain’s “reward network”, particularly the ventromedial prefrontal cortex [16]. While the authors of the studies included in this scoping review were generally optimistic about the potential for art therapy and art making as a non-pharmacologic strategies for managing pain and emotional distress in cancer survivors, all brought up limitations in study design, small sample sizes, interruptions in scheduling, and variations in the types of interventions utilized, which limited their ability to draw any definitive conclusions. These limitations underscore the need for more rigorous and generalizable studies to establish the efficacy and scalability of art therapy and art-making in oncology care.

In addition, the distinction between art therapy and art making remains ambiguous due to different credentialing practices both within the US and globally. Within the scientific literature, the terms are sometimes conflated or used interchangeably [38]. Art therapy certification is limited to certain parts of the world (the US, Canada, Australia, the United Kingdom, and parts of Europe), although pilot programs have been launched in parts of Asia [39]. Asian studies included in this review were referred to as either art therapy or art making; however, most were not delivered by certified art therapists [25,35]. The exception is the MBAT study by Jang and colleagues [24], which took place in Korea, but was delivered by a certified art therapist.

Several programs that took place stateside were not delivered by certified art therapists [17,18,23,26,27,30,31,32,34]. Among the studies that utilized certified art therapists [4,5,6,8,20,21,22,24,28,29,33], there were many variations in the therapeutic approaches they used, whether the art therapy was delivered individually or in group settings, whether it was the sole intervention or if it was combined with other psychotherapeutic modalities, and the types of materials employed, which, in one case [5], included both visual arts and music interventions.

Part of this diversity is reflected in the history of art therapy, dating back to the 1940s, which evolved from two very different theoretical frameworks: a psychoanalytic approach, as proposed by Margaret Naumburg, and an art for art’s sake approach developed at about the same time by Mary Huntoon [12]. Today, art therapy takes many forms, which can be conceptualized as a continuum between those two endpoints. Among the more psychoanalytic approaches are Naumburg’s dynamically oriented art therapy (or art psychotherapy), archetypal art therapy [40], Gestalt art therapy [41], and the Expressive Therapies Continuum or ETC [42], to behavioral approaches (Cognitive Behavioral Art Therapy) [43], neuroscientific approaches, spiritual modalities, and the open studio approach [44], which often takes place in community settings and comes close to Huntoon’s original vision, stressing the therapeutic value inherent in any creative expression. This heterogeneity is reflected in research on the subject, with some studies focusing on art making, and others on various forms of art therapy, ranging from open studio to more analytic approaches.

This leaves open the question of assessing the effectiveness of a particular art therapy or art making intervention to address cancer pain and emotional distress. While the quantitative measures utilized in determining outcomes for these studies are well-accepted and validated, a protocol suggesting a specific measure would make it easier to compare outcomes and draw conclusions regarding efficacy. A brief measure, such as the PEG 3-item questionnaire [45], puts relatively low burden on patients, but can yield important information about the levels of subjectively rated pain intensity, its impact on overall function, and quality of life. Combining this with well-curated interviews could create the basis for richer literature on the use of art therapy and art making for addressing cancer-related pain and distress, which is important for guiding future interventions.

The diversity of art therapeutic approaches contributes to their versatility: they can be adapted for a variety of clinical scenarios and implemented in settings ranging from acute care to the community. The term “art therapy” must be clearly defined, and if possible, the type of art therapy employed (e.g., psychotherapeutic versus open studio) should be included. It will be important to assess the relative evidence for these interventions as relates to cancer staging and other therapeutic modalities, particularly medications. Finally, studies should address the acceptability of art therapy and art making within clinical settings, as this has a significant impact on whether such modalities will be utilized with oncology patients.

### 6.1. Practical Implications

The authors propose the following considerations for individuals interested in pursuing art therapy or art making interventions in inpatient or outpatient settings, based on the studies reviewed:

1. Initial engagement can be challenging, particularly in inpatient settings where cancer survivors are emotionally fragile. It is important to stress that this is not meant to be skill training, but rather an opportunity for self-expression [8,29]. In general, women may be more amenable to art making than men because of earlier and more extensive cultural awareness [34].
2. Inpatients undergoing aggressive treatments (e.g., bone and marrow transplants) are immunocompromised. Therefore, attention to a sanitary environment is essential [27]. In addition, these individuals generally have lower energy levels, so keeping sessions brief can avoid overstimulating the patients.
3. Attention diversion typically lasts as long as the intervention. Therefore, it may be advisable to continue art making or art therapy sessions throughout active treatment and, if possible, give cancer survivors opportunities for art making in follow-up encounters in primary care [25].
4. Creating art can give color and form to emotions. Therefore, art making may be a good method for cancer survivors to process difficult emotions related to anger about a diagnosis and body estrangement [32].
5. For some cancer survivors, art making in the form of gifts to friends and providers can be a form of reciprocal care [32].
6. Art making has been shown to shift attention away from the inpatient environment in palliative care to address depression and anxiety triggered by one’s surroundings [33].
7. Art making is a form of storytelling, which can be a powerful method of communication for Indigenous cancer survivors [18].

### 6.2. Limitations

There exists a limitation in that only English-language databases were searched, and gray literature was not included. Gray literature might include patient narratives regarding their art making or art therapy experiences. Evidence for the efficacy of art making and art therapy in the extracted studies is limited by the heterogeneity of approaches and settings; generally small sample sizes, which reduce statistical power; and, in some cases, study designs lacking control groups. Studies took place over different time frames, lasting from documentation of single art therapy sessions to long-term art making. To compare outcomes from these studies in terms of short or long-term benefits for patients is not possible. This is not to suggest that investigation of these complementary alternative approaches lacks value, but rather that it will be important to update information as new studies become available.

## 7. Conclusions

The studies reviewed show mixed outcomes as to the value of art therapy and art making interventions for controlling pain, stress, anxiety, and depression among cancer survivors. However, the authors of this review are optimistic regarding the potential for utilizing these modalities as part of a multi-modal strategy for improving quality of life among cancer survivors. Medications, while a valuable resource for controlling pain and, in some cases, affective disorders, have well-known limitations including tolerance, habituation, and drug interactions. Because both chronic pain and emotional distress can be long-term problems for cancer survivors, non-pharmacologic strategies, particularly those that are low-cost and can be delivered in a variety of settings, can be valuable assets. In addition, art creation has the potential for helping cancer survivors to process feelings of anger and hopelessness and, in some cases, find new meaning in their lives. Neuroscience is in the early stages of investigating the mechanisms underlying our human need to express ourselves visually. Future work should utilize advances in neuroimaging technology to investigate the therapeutic value of art making in chronic pain management. For cancer survivors, such studies could have significant implications for both short and long-term management of these symptoms.

## Figures and Tables

**Table 1:** Research design, populations, and evaluation measures for included studies.

Authors, Publication Date	Participants	Study Design	Evaluation Measures
Collette et al., 2022 [20]	540 cancer survivors, relatives, and health care providers from 14 studies.	Mapping review	Surveys, semi-structured interviews, ESAS, BSI-18, PG-13, HADS, POMS, SF-36, PROMIS, aesthetic dimensions assessment.
Egberg Thyme et al., 2009 [21]	41 breast cancer survivors.	Randomized controlled trial	Symptom Check List-90, Structural Analysis of Social Behavior.
Elimimian et al., 2020 [22]	50 cancer survivors with breast cancer, gastrointestinal cancers, and hematologic malignancies.	Single arm pilot	Visual analog scales for pain and depression.
Ennis et al., 2016 [23]	421 male and female cancer survivors with various diagnoses.	Integrative review	Thematic analysis. Quantitative measures not listed.
Hammond et al., 2022 [17]	6 caregivers of cancer survivors within a Mohawk First Nation in Canada.	Observational	Interviews with community members.
Hodge et al., 2022 [18]	17 Native American female cancer survivors.	Observational	Drawings by participants used to evaluate pain. No pain scales were utilized.
Jang et al., 2016 [24]	24 breast cancer survivors.	Randomized controlled trial	EORTCQLQ-C30.
Junhui et al., 2024 [25]	2,050 cancer survivors with various diagnoses.	Scoping review	MAT, SAS, SDS, QOL, PANAS, VAS, STAI, HHI, EORTCQLQ-C30, FLIE, HADS, CRI, SASB, SCL-90, POMS, BFI, FACT-G, Distress Thermometer.
Kelley et al., 2025 [26]	54 cancer survivors.	Mixed methods	Semi-structured interviews.
Kievisiene et al., 2020 [5]	519 breast cancer survivors.	Systematic review	CRI, EACS, ESI-R, POMS, WHOQOL-BREF, EORTC-QLQ-BR23, SASB, SCL-90, SF, BSI, PAI, AE, CES-D, BCPT.
Lawson et al., 2012 [27]	20 oncology inpatients undergoing bone marrow transplantation.	Mixed methods	Therapy-Related Symptom Checklist, Spielberger State–Trait Anxiety Checklist.
Lee et al., 2017 [28]	24 cancer survivors undergoing radiotherapy.	Mixed methods	ESAS, HADS, HDRS.
Lefèvre et al., 2016 [6]	22 (16 female and 6 male) cancer survivors.	Mixed methods	ESAS
Luzzatto et al., 2003 [29]	70 cancer survivors (60 female, 10 male).	Observational	Body Outline art therapy used to communicate pain ratings.
Mollaoğlu et al., 2024 [7]	60 cancer survivors (female).	Randomized controlled trial	FACT-G.
Monti et al., 2013 [30]	260 breast cancer survivors.	Randomized controlled trial	SCL-90, SF-36.
Nainis et al., 2006 [8]	50 cancer survivors at an inpatient oncology clinic, various diagnoses.	Quasi-experimental	ESAS, STAI-S.
Puig et al., 2006 [31]	39 Stage I and II breast cancer survivors.	Mixed methods pilot	POMS, EACS, ESI-R.
Reynolds & Lim, 2007 [32]	12 female cancer survivors living in the community.	Observational	Participant interviews.
Rhondali et al., 2012 [33]	12 female cancer survivors with metastasis.	Mixed methods	Semi-qualitative patient interviews, ESAS.
Saw et al., 2018 [34]	31 (21 female and 10 male) cancer survivors with hematologic malignancies.	Mixed methods pilot	Visual analog scale for pain, STAI, Positive and Negative Affect Schedule.
Soo Kim et al., 2018 [35]	697 cancer survivors with various diagnoses.	Systematic review	ESAS, EORTC-QLQ-30, FACIT-Sp, SF-36, WHOQOL-BREF, HADS.
Zhou et al., 2023 [14]	721 cancer survivors, male (n = 4); female (n = 717).	Systematic review and meta-analysis	SCL-90, SF-36, WHOQOL-BREF, EORTC-QLQ-BR23, EORTCQLQ-C30, POMS, SAS.

Note: For explanation of rating scale abbreviations, please see Appendix B.

**Table 2:** Art therapy and art making interventions for cancer-related pain and emotional distress.

Authors, Publication Date	Type of Art Therapy/Art Making	Duration of Therapy	Types of Providers	Outcomes
Collette et al., 2022 [20]	Painting, drawing, collage, sculpting, and photography.	Multiple sessions lasting 30–150 min, up to 8 weeks.	Art therapists.	Statistically significant improvements in pain, emotional distress and QoL.
Egberg Thyme et al., 2009 [21]	Drawing and painting using a variety of media.	5 sessions, duration not specified.	Art therapists.	Statistically significant reductions in depression, anxiety, and somatic symptoms.
Elimimian et al., 2020 [22]	Drawing, painting, ceramics, and collage.	Multiple sessions (number and duration not specified).	Art therapists.	Significant reductions in pain, anxiety, and emotional distress.
Ennis et al., 2016 [23]	Tile painting, mixed media, collage, mosaic, and craft making.	Multiple sessions lasting 60–120 min, up to 6 months.	Art facilitators.	Distraction from pain, improved coping and self-efficacy.
Hammond et al., 2022 [17]	Digital storytelling by caretakers.	2 years.	Academic and community researchers.	Caregivers expressed the need for emotional support from others in the community.
Hodge et al., 2022 [18]	Drawings of cancer-related pain.	Number of sessions and duration not specified.	Researchers and clinicians.	Drawing helped Indigenous cancer survivors communicate more easily about pain than using English language.
Jang et al., 2016 [24]	Mindfulness-Based Art Therapy (MBAT).	12 sessions, 45 min each.	Therapists trained in Korean Mindfulness-Based Stress Reduction (K MBSR).	Reductions in pain trending towards significance. Significant reductions in depression and anxiety.
Junhui et al., 2024 [25]	Drawing therapy.	25–150 min, up to 12 weeks.	Nurses not trained in art therapy and art therapists.	Improvements in pain, anxiety, depression and quality of life.
Kelley et al., 2025 [26]	Watercolor painting.	90 min, single session.	Provider not specified. Intervention included art instruction.	Significant reductions in subjective pain and stress ratings.
Kievisiene et al., 2020 [5]	9 RCTs of art therapy and 11 of music therapy. Art therapy included MBAT, psychoanalytic art therapy, and mandala coloring.	Multiple sessions, ranging from 45–150 min and up to 12 weeks.	Art therapists and art facilitators.	Small effect for physical symptoms including pain. Significant reductions in anxiety and depression and increased QoL.
Lawson et al., 2012 [27]	Ceramic tile painting.	40-60 min. Number of sessions not specified.	Occupational therapy faculty and students.	Reduction in stress and anxiety compared to controls.
Lee et al., 2017 [28]	Famous painting viewing and creative artwork generation in relation to the famous paintings.	30 min/twice a week for 8 weeks.	Art therapists.	Improvements in depression and anxiety, but no improvements for subjective pain.
Lefèvre et al., 2016 [6]	Painting, drawing, modeling, photography, and sculpture.	1–10, 60-min sessions.	Art therapists	Statistically significant reductions in pain, anxiety, fatigue, and depression.
Luzzatto et al., 2003 [29]	Body Outline drawing exercise.	Not specified.	Art therapists.	Patients used the body outline template to communicate about their pain, emotions, and search for spirituality.
Mollaoğlu et al., 2024 [7]	Marbling and ney music.	Art therapy following biweekly chemotherapy sessions for treatment group, with sessions lasting 30 min.	Art therapists.	Treatment group reported significant decreases in pain, nausea, and anxiety, while the control group reported no statistically significant changes.
Monti et al., 2013 [30]	MBAT.	Multiple sessions, duration not specified.	Art therapists.	Significant reductions in somatic symptoms and stress, with improvements lasting for up to 6 months.
Nainis et al., 2006 [8]	Art therapy using a variety of media.	Single sessions.	Art therapist.	Significant improvements in pain, fatigue, nausea, depression, and anxiety.
Puig et al., 2006, [31]	Semi-structured art therapy sessions using drawing and painting.	90 min, 4 weeks.	Art therapists.	Significant reductions in depression and anxiety, not significant for somatic symptoms.
Reynolds & Lim, 2007 [32]	Independent art making by cancer survivors: collage, pottery, painting, and card making.	Not specified.	Art facilitators.	Reductions in experienced somatic symptoms, improved self-efficacy.
Rhondali et al., 2012 [33]	Painting.	1 h sessions, duration of therapy not specified.	Art therapist.	Statistically significant improvements in pain, fatigue, depression, and anxiety.
Saw et al., 2018 [34]	Watercolors, colored pencil drawings, and ceramics.	30 min minimum, some sessions lasted longer.	Art educator in conjunction with clinicians.	Improvements in self-reported mood, anxiety, and pain.
Soo Kim et al., 2018 [35]	Painting, drawing, collage, craft, photography, meditation, music, and creative movement.	60–150 min, up to 22 sessions.	Not specified.	Improvements in pain (not statistically significant) and mixed results for quality of life.
Zhou et al., 2023 [14]	MBAT, individual art therapy, group art therapy, and individual painting.	45–60 min, single session to 8 weeks.	Art therapists.	No significant difference in somatic symptoms, significant improvements in QoL and depression.

## Data Availability

No new data were created or analyzed in this study. Data sharing is not applicable to this article.

## Supplementary Materials

The following supporting information can be downloaded at: https://www.mdpi.com/article/10.3390/ijerph22121877/s1, File S1: Preferred Reporting Items for Systematic reviews and Meta-Analyses extension for Scoping Reviews (PRISMA-ScR) Checklist [46].

## Appendix A. Search String, Scopus

The following search string generated 42 results in Scopus, February, 2024: (TITLE-ABS-KEY (“cancer pain”) AND TITLE-ABS-KEY (“art therapy” OR “art making”).

## Appendix B. Rating Scale Abbreviations

**Abbreviation**	**Name of Scale**
AE	Acceptance of Emotions Scale
BCPT	Breast Cancer Prevention Trial Scale
BFI	Big Five Inventory
BSI	Brief Symptom Inventory
CES-D	Center for Epidemiologic Studies Depression Scale
CRI	Coping Response Inventory
EACS	Emotional Approach Coping Scale
EORTCQLQ	European Organization for Research and Treatment of Cancer Quality of Life Questionnaire
ESAS	Edmonton Symptom Assessment Scale
ESI-R	Expressions of Spirituality Inventory Review
FACIT	Functional Assessment of Chronic Illness Therapy Scale
FACT-6	Functional Assessment Cancer Therapy Scale
FLIE	Functional Living Index Emesis Scale
HADS	Hospital Anxiety Depression Scale
HDRS	Hamilton Depression Rating Scale
HHI	Herth Hope Index
MAT	Medication Assessment for Cancer Pain Management
PAI	Personality Assessment Inventory
PANAS	Positive And Negative Affect Scale
PG-13	Prolonged Grief Disorder Scale-13
POMS	Profile of Mood States
PROMIS	Patient Reported Outcomes Measurement Information Scales
SAS, SASB	Self-Rating Anxiety Scale (SAS)—Brief
SF-36	36-Item Short Form Health Survey
SCL-90	Symptom Check List 90
STAI	Spielberger State–Trait Anxiety Index
VAS	Visual Analog Scale for Pain
